# Folates, folic acid and preconception care – a review

**DOI:** 10.1177/2054270420980875

**Published:** 2021-05-13

**Authors:** Simon H House, John AA Nichols, Sarah Rae

**Affiliations:** 1 Royal Society of Medicine, London W1G 0AE, UK; 2 Mother & Child Foundation, Portsmouth PO5 2DS, UK; 3 Department of Nutritional Sciences, 3660 University of Surrey, Guildford GU2 7XH, UK; 4 1578 Bedford Hospital NHS Trust, Bedfordshire MK42 9DJ, UK

**Keywords:** brain stem, cerebellum, clinical, clinical genetics, genetics, neurology, nutrition and metabolism, obstetrics and gynaecology, other nutrition and metabolism, reproductive medicine

## Abstract

The link between folate deficiency and congenital spina bifida defects was first suggested in the 1960s. Although the prevention of these defects by preconception folic acid supplementation was confirmed in a large multi-centre controlled trial in 1991, its subsequent implementation as health education advice has made very little difference. North America’s policy of folic acid fortification of flour and bread has had a beneficial impact. No European country has implemented fortification due to concern over possible adverse effects on older subjects, but a recent review shows these to be largely hypothetical and far outweighed by beneficial effects. Recent research by Menezo et al. has, however, shown that folic acid is ineffective for some women with severe fertility problems including recurrent miscarriage and failed in vitro fertilisation. There is a genetically determined bottleneck (677TT) in their folate metabolism that can be successfully overridden by going straight to the next step in the metabolic pathway and taking 5-methylytetrahydrofolate, as a preconception supplement. Menezo suggests that all women with fertility problems should be tested for 677TT. If fortification of flour and bread is to be implemented in the UK, there should be monitoring for possible adverse effects including the incidence of colorectal cancers and cognitive decline. In conclusion, whilst there are concerns that fortification could have a detrimental effect on these conditions, there is sound evidence that it would have much greater beneficial effects.

## Background

**Figure 1.**
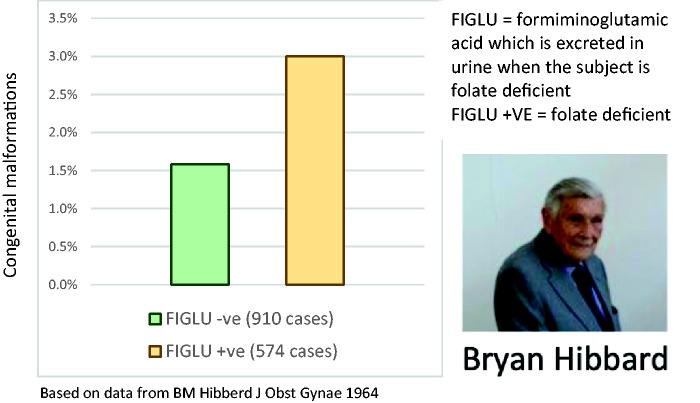
Bryan Hibbard’s breakthrough paper showing a link between folate deficiency and congenital malformations.

The folic acid story began in 1964 when Bryan Hibbard was a young consultant obstetrician in Liverpool researching the causes of placental abruption. He found a link with folate deficiency and also noted a higher incidence of congenital malformations in folate (vitamin B9)-deficient subjects (Figure 1).^1^

Leck^2^ and Smithells et al.^3^ then suggested that folate deficiency had a causal link with congenital neural tube defect (spina bifida) malformations. They found that the commonest is spina bifida with meningocoele. The damage to nerve tissue incorporated in this congenital herniation of the cauda equina cannot be repaired by the surgical correction of the menigocoele and the child will almost certainly survive to be a lifelong paraplegic. Far more serious is anencephaly when the failure of closure of the neural tube is at the upper end and the back of the head is an open red mess with almost complete failure of development of the brain. These infants will probably expire within 1–2 h of birth. Two lesser versions of upper spina bifida malformation, which can still be associated with severe disability, are hydrocephalus and microcephaly. Subsequent research indicated that preconception folic acid significantly reduces the risk of neural tube defect malformations.^4,5^ Folic acid is a convenient chemically stable synthetic version of folate that is easily metabolised to folate, whereas dietary folate degrades easily. This paper reviews: the limited success of preconception folic acid as a public health strategy^6,7^ despite the success of controlled trials of folic acid;^4,5^ the case for folic acid fortification of bread and flour; and the results of recent research that suggest there are limitations to recommending folic acid in some clinical circumstances.

## Method

Data were gathered through primary care research in the area of periconceptional health, through attending meetings on folic acid fortification as a representative of the Royal College of General Practitioners and through searching the internet (mainly Medline Plus and Google Scholar) using the search terms folate, folic acid and neural tube defect. Significantly, this paper was inspired by involvement in organising the speakers for a day conference at the Royal Society of Medicine entitled ‘Folic Acid Fortification and Preconception Care’ in February 2020.

## Building the case for folic acid fortification of bread and flour

Following the work of Hibbard and his Liverpool paediatrician colleague, RW Smithells, further observational and epidemiological evidence accumulated on folate deficiency and neural tube defects and at the same time the metabolic pathways of folate were delineated. Finally, the MRC organised the definitive randomised control trial, which was masterminded by Nicholas Wald of the Wolfson Institute of Preventive Medicine.^4^ This is a good example of the three strands of nutrition science combining to make an impact: epidemiology/observational research,^1–3^ biochemistry^8–10^ and large-scale trials (Smithells^11^ and MRC trial^4^).

**Figure 2.**
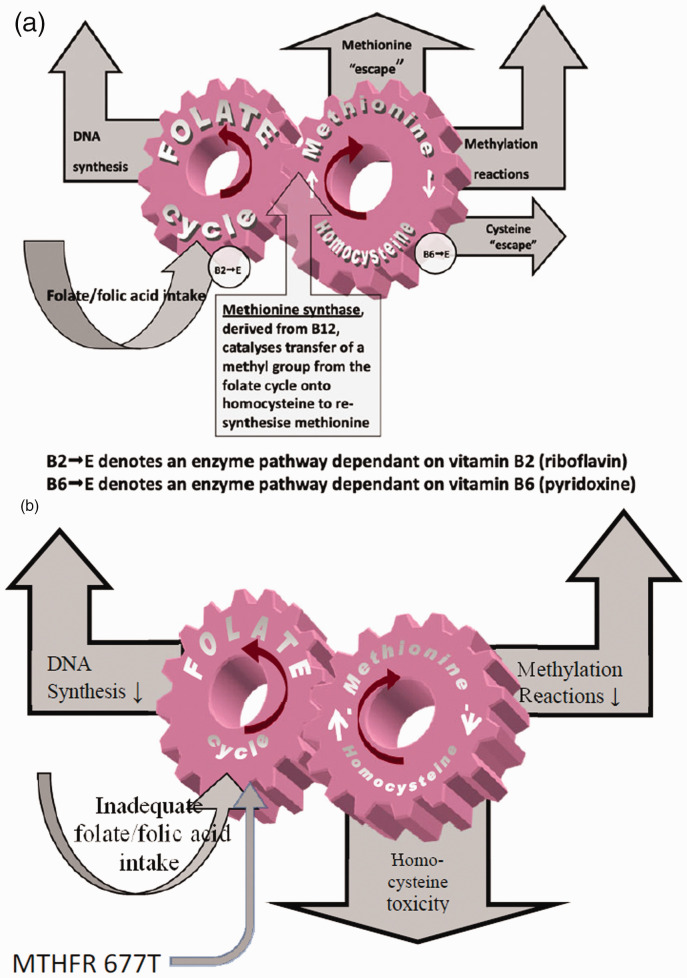
(a) Simplified version of the folate–methionine–homocysteine cycle illustrating the role of vitamins B2 (riboflavin), B6 (pyridoxine) and B12 (cobalamin) in modulating the cycle and directly or indirectly lowering homocysteine burden. (b) Simplified version of changes to folate metabolism in folate deficiency. Inadequate dietary folate or a genetic variant of the methylene tetrahydrofolate reductase (MTHFR) gene (or a combination of both) can give rise to congenital malformations – especially neural tube defects.

The stability of this metabolic pathway, therefore, depends on sufficiency of four B vitamins, but especially vitamin B9 – folate. As long as the wheels keep turning there is a constant supply of DNA and methyl groups to fuel many key metabolic pathways that are especially important in the development of the embryo and foetus (Figure 2(a)). Most important of all is methylation of DNA as the basis for a significant component of epigenetic gene switching and control of gene expression relevant to the development of the foetus.^12^ If the input of folate into this metabolic pathway is curtailed due to dietary insufficiency, the ‘wheels’ slow down, DNA synthesis and many other metabolic pathways are compromised and the toxic amino acid homocysteine builds up. This produces two adverse consequences as both the slowing of the synthetic pathways and the toxicity of homocysteine have an adverse effect on fetal development. Furthermore, if the subject is homozygous for the 677T variant of the gene for the enzyme methylene tetrahydrofolate reductase this will slow introduction of folate into the folate cycle side of the metabolic process, slow down the synthetic pathways and push up blood homocysteine levels (Figure 2(b)). Therefore, if a subject is both homozygous for 677T gene (i.e. 677TT) and has a folate deficient diet, there are almost bound to be adverse consequences. A subject who is homozygous for the normal speed 677C variant is much less sensitive to folate deficiency. There are several steps in the metabolic pathway from folic acid to the version of folate that is metabolised by methylene tetrahydrofolate reductase to L-5-methyltetrahydrofolate, which fuels the folate side of the folate–methionine–homocysteine cycle. The homozygous 677TT combination creates a bottleneck that reduces the rate of production of L-5-methyltetrahydrofolate. In some clinical circumstances, bypassing this bottleneck by treating the patient with L-5-methyltetrahydrofolate has been shown to be more effective than simply increasing the dose of folic acid.^13–15^

The data from a 2002 UK government population survey showed that the mean dietary intake of folate for all women was 250 µg/day which is below the recommended intake of 300 µg/day.^16^ This served to confirm the government policy of encouraging women to take a folic acid supplement starting before conception. Paradoxically, the lowest mean folate intake (229 µg/day) was seen in women aged 19–24 when fertility is at a maximum. The problem of folate deficiency starts in childhood. A 2004 survey of 5990 German schoolchildren^17^ showed that for the 13–18 years age bracket, the only way to reach the minimum recommended intake for folate is to have a diet that includes food and drink fortified with folic acid and to take a vitamin supplement that included folic acid. Today’s 18-year-old girls are tomorrow’s mothers and only education in health and diet (particularly in relation to conception) in schools can improve the quality of these children’s diets.

**Figure 3.**
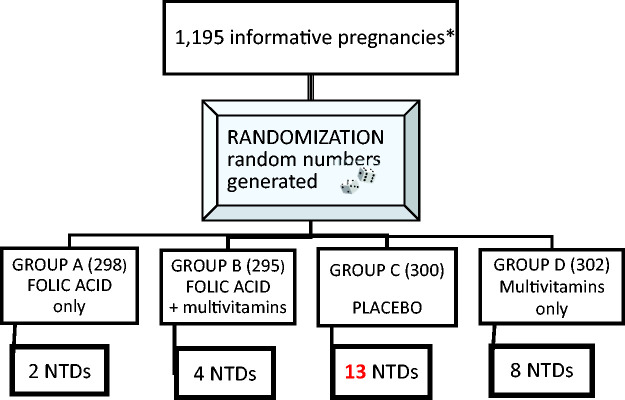
1991 MRC trial on prevention of neural tube defects (NTDs) with folic acid 4000 mcg daily with/without multivitamins versus placebo with/without multivitamins.^4^ *An informative pregnancy was one in which the fetus or infant was known to have or not have a neural tube defect by the time the trial was stopped.

The 1991 Medical Research Council trial explored the value of giving a folic acid supplement for prevention of neural tube defects. A total of 1871 women who had already had a neural tube defect pregnancy were recruited from seven countries. There was considerable discussion at the time as to whether it was ethical to give a placebo to the 454 women randomised to the placebo arm considering the weight of evidence that folate deficiency caused neural tube defect malformations.^4^ The results shown (Figure 3) represent the stage at which the trial was terminated on ethical grounds and all subjects were switched to folic acid 4000 µg daily. However, Wald calculated that, taking confounding factors into consideration (mainly poor compliance in some subjects in the two folic acid arms) there was an 83% protection against neural tube defects in women taking preconception folic acid 4000 µg daily. This implies that 17% are not helped by folic acid and there may be genetic factors involved or some subjects may have been B12 deficient.^18^ Subsequently, UK government policy was to advise a smaller preconception dose of 400 µg daily for most women but 4000 µg for women who have had a neural tube defect pregnancy and to women taking an antifolate drug such as epanutin and women with diabetes. Wald maintains that, ideally, all women should be taking 4000 µg daily for optimal effect. Although the incidence of neural tube defect malformations in the UK has decreased since 1994, we are still a long way from perfection. All women are screened for fetal congenital malformation by ultrasound scan and offered a termination of pregnancy if this shows a neural tube defect malformation – a policy referred to as selective abortion.

Despite government advice on preconception folic acid, over 50% of European women fail to follow this advice.^6,7^ They may start taking folic acid when they know they are pregnant but this is too late. The closure of the neural tube is either complete by then or else it has failed completely with no possibility of correction. Another problem is the vague nature of UK government advice as to how long before the planned conception folic acid should be started. Some experts say ‘just when you decide to start trying for a baby’ other insist that it should be three months before conception.^19^

Our research at the University of Surrey shows a relatively high adherence to government guidelines with 59% of local women taking either folic acid or a preconception supplement that includes folic acid 400 µg. About 24% of women had pregnancies that were unplanned but not unwanted. The UK national average for pregnancies that are unplanned but not unwanted is 40%, which means that 40% of pregnancies will never be able to benefit from preconception folic acid. The only certain way to reach these women and their babies is folic acid fortification of flour and bread as recommended by the UK government Scientific Advisory Committee on Nutrition. Politicians have commented, however, that implementing this recommendation is ‘low on our list of priorities’. Based on my own research,^20,21^ data from North America^22^ and data from the Wolfson Institute,^23^ mandatory fortification would result in 300 healthy infants per year being born that are currently mostly terminated for neural tube defect following antenatal screening by ultrasound. However, folic acid fortification remains a little controversial (Table 1) and was halted in 2007 due to concerns that it might increase the risk of colorectal cancer.^24^

**Table 1.** Evidence for benefits and risks of folic acid fortification.

Favours fortification	Against fortification
300 normal births/year – currently, mainly lost by selective abortions for neural tube defects.	Unmetabolised folic acid may be a problem.
Improved folate status relevant to reduced risk of autism, leukemia and childhood cancers.	High folate status in older subjects may accelerate cancers.
Evidence from North America for reduction of cancers.	High folate status may exacerbate B12 deficiency.
Evidence from North America for 5% reduction of stroke and general benefits of lowering blood homocysteine levels.	Recent evidence that folate is better than folic acid for infertility and fetal brain development.^12,15^

### Arguments for and against UK folic acid fortification

**Figure 4.**
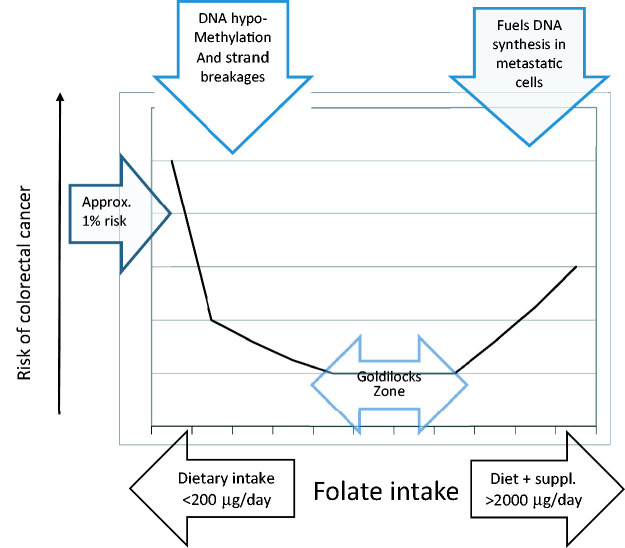
Folate intake and risk of colorectal cancer.

Table 1 shows the pros and cons of folic acid fortification. Generally speaking, the evidence against fortification is weaker than the evidence for benefits. For instance, the dangers of unmetabolised surplus folic acid, which has been identified on blood samples, are largely hypothetical.^25^ There should be very little danger that folate status from fortification will reach high enough levels to exacerbate B12 deficiency and general medical practitioners (family doctors) should be testing for B12 deficiency in older subjects almost routinely. One danger is that cancer patients may reach a high folate status mainly because they are taking a vitamin supplement that includes folic acid on top of intake from folic acid fortified food. This would be especially undesirable in patients taking folate antagonists such as methotrexate or 5-flourouracil for their cancer when they should be advised to avoid folic acid containing multivitamins and to look on the supermarket shelves for unfortified bread, which should be made available for such patients and for anyone who wants to take unfortified bread on ideological grounds. The evidence for a link between high folic acid intake causing an increased risk of colorectal cancer is based on a supplementation trial comparing folic acid 1000 µg daily with placebo.^26^ However, there is a higher risk of colorectal cancer from folate deficiency than from folic acid excess as illustrated by current data. As with most nutrients and micronutrients, there is a wide range of safe intake which can be thought of as ‘the goldilocks zone’ (Figure 4). With folate deficiency, there is DNA hypomethylation and strand breakages that can lead to cancerous mutations. This is well documented.^27^ There is some evidence that folate status at the highest intake fuels DNA synthesis in metastatic cells and accelerates cancer growth. There was, in fact, a brief but limited increase in the incidence of colorectal cancers in the United States after folic acid fortification. However, further follow-up of colorectal cancers in the United States does indeed show that the rise in cases at the start of the decade was shown to be brief and limited. There has been a steady decline in cases throughout the rest of the decade and this coincides with a rise in the average total intake of folates + folic acid.^28^

Recent evidence from two sources, Servy and Menezo^13^ and Jimenez et al.,^15^ seem to show the limitations of folic acid in some clinical circumstances. Menezo has shown that women with severe fertility problems (including failed in vitro fertilisation, recurrent miscarriage and stillbirth) and the homozygous 677TT genetic status do not benefit from high dose folic acid. He has shown good results from bypassing the 677TT bottleneck by giving 5-methyltetrhydrofolate as a preconception supplement. Menezo has suggested that all women with fertility problems and their partners should be tested for the MTHFR667 gene variants and treated with 5-methyltetrhydrofolate when appropriate and that biomarkers including blood homocysteine levels indicate that 5-methyltetrhydrofolate should be given for at least three months before a planned conception. Perhaps, the responsibility for testing for MTHFR667 gene variants should be included in the routine workup by general medical practitioners prior to referral to a fertility clinic.

Assuming that the UK’s mandatory folic acid fortification goes ahead, we should bear in mind some of the genuine concerns that have been noted (Table 1). A well-organised National Health Service and government research funding should make it possible to monitor the outcome of fortification. There are two options:

1. A very large scale but superficial survey recording the folic acid levels in bread and flour and pregnancy outcomes and taking random blood samples for folate and B12.
2. Smaller in-depth survey carried out at three centres chosen as representative of the UK population. This would include monitoring for cognitive decline and brain shrinkage in relation to changes in blood levels of folate and B12.

If the level of folic acid in bread and flour is in the safe ‘goldilocks zone’ then the incidence of colorectal cancers should decline rather than increase. The second option of in-depth monitoring is based on a large body of research on a variety of topics relevant to folate status including Alzheimer’s disease,^29^ autism^30^ and nutritional aspects of cancer^27,28^ (Box 1).

**Box 1.** Suggested monitoring criteria for option 2

• Monitor folic acid levels in bread and flour
• Monitoring of public awareness of fortification policy and alternative unfortified options for cancer patients (especially those on antifolates for cancers)
• B12 status in subjects aged >65 years
• Blood folate levels
• Neural tube defect and other congenital malformations and miscarriages
• Incidence and ages of new cases of autism and statementing
• Monitor cognitive decline in a sample of older subjects
• Random MRI brain scans in 65–80 years age group to monitor brain shrinkage
• Incidence of new cases of colorectal cancers

## Conclusions

The current policy of advising women to plan pregnancies and to take a daily dose of 400 µg of folic acid before conception has largely failed. Half of women with planned pregnancies ignore this advice. Nearly half of women have unplanned pregnancies that they decide to continue. Therefore, they never have the opportunity of taking a preconception supplement. Going on the experience of other countries who have implemented mandatory folic acid fortification of bread and flour, at least 300 UK babies a year would be saved, most of whom are currently aborted. However, folic acid is not a panacea. There are clinical circumstances including fertility problems when by passing the methylene tetrahydrofolate reductase bottleneck by treating with 5-methyltetrhydrofolate is more appropriate. These subjects are unlikely to get any benefit from preconception folic acid or from folic acid fortification. There are many other aspects of periconceptional nutrition and the environment (i.e. maternal stress, dietary selenium, omega-3 and iodine) that are beyond the scope of this review. When mandatory folic acid fortification is finally implemented, there should be a detailed monitoring programme in place that should include monitoring of the incidence of Alzheimer’s disease and colorectal cancers.
